# Insights from multigene analysis: first report of a Southeast Asian Mosquito, *Aedes* (*Mucidus*) *laniger* (Diptera: Culicidae) on Jeju Island from Korea

**DOI:** 10.1186/s13071-024-06373-8

**Published:** 2024-09-12

**Authors:** Woo Jun Bang, Ara Seol, Seunggwan Shin

**Affiliations:** 1 School of Biological Sciences, Seoul National University, Seoul, 08826 Republic of Korea; 2 Comparative Medicine Disease Research Center, Seoul National University, Seoul, 08826 Republic of Korea; 3 Warm Temperate and Subtropical Forest Research Center, National Institute of Forest Science, Jeju, 63582 Republic of Korea

**Keywords:** *Aedes* laniger, Culicidae, Non-native mosquito, Jeju Island, Species distribution modelling

## Abstract

**Background:**

Certain mosquitoes are known as dominant vectors worldwide, and transmit infectious diseases. The expansion of mosquito habitats due to climate change and increased human activities poses a significant health threat by facilitating the spread of various non-native infectious diseases. This study focused on the detection of the Southeast Asian mosquito species, *Aedes* (*Mucidus*) *laniger* (Wiedemann, 1820) on Jeju Island, the southernmost region of the Republic of Korea (ROK), highlighting the potential risks associated with the spread of vector-borne diseases, particularly emphasizing the elevated likelihood of invasion by Southeast Asian mosquitoes.

**Methods:**

Field surveys were conducted in August 2023 on Jeju Island. Adult mosquitoes were collected using BG-sentinel traps and identified to the species level using taxonomic keys. Morphological and molecular analyses were employed to confirm species designations. Molecular data, including mitochondrial and nuclear genes, were used for phylogenetic analysis, which was performed to compare and identify among recorded subgenera in ROK. Species distribution modeling for *Ae*. *laniger* was performed to predict potential habitats using R package ‘BIOMOD2’.

**Results:**

The two specimens of *Ae*. *laniger* were collected for the first time on Jeju Island. Morphological and molecular analyses confirmed the identity of this species within the subgenus *Mucidus* and validated the first record of this species in the ROK. We employed a simple multigene phylogenetic analysis to confirm a new mosquito record at the genus and subgenus levels, finally validating the consistency between morphological identification and molecular phylogenetic outcomes. Furthermore, we have updated the taxonomic keys for the genus *Aedes* in the ROK, and revised mosquito lists for Jeju Island, incorporating the inclusion of *Ae*. *laniger*. On the basis of species distribution modeling, the area of suitable habitat for *Ae*. *laniger* is expected to expand due to climate change, but this change did not appear to be meaningful in East Asia.

**Conclusions:**

This case offers the first report of the Southeast Asian mosquito, *Ae*. *laniger*, in the ROK. The detection of this species on Jeju Island suggests the potential establishment of a breeding population their habitat and raises concerns about further expansion into the Korean Peninsula. Considering the annual occurrence of mosquito-borne disease cases in the Southeast Asia, it is essential to conduct monitoring not only in Jeju Island, where *Ae*. *laniger* has been identified, but also across the entire Korean Peninsula.

**Graphical Abstract:**

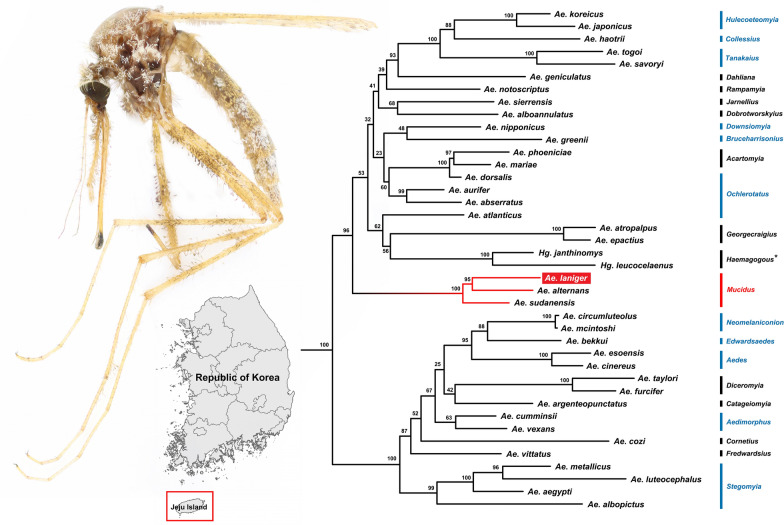

**Supplementary Information:**

The online version contains supplementary material available at 10.1186/s13071-024-06373-8.

## Background

Certain species of mosquitoes are dominant vectors worldwide and drive the spread of infectious diseases through their well-developed piercing mouthparts during the blood-feeding process [1]. Mosquito species can transmit various diseases, such as malaria and dengue fever, leading to more than 700,000 deaths worldwide annually [2]. Factors such as climate change, increased anthropogenic activities and material exchanges among countries have led to recent expansions in mosquito habitats [3–6].

Mosquitoes that are spreading globally predominantly belong to the genus *Aedes*, and the majority of these species have a strong ability to adapt to changing environments [7, 8]. In addition, some species have also been listed on the Invasive Species Specialist Group [8, 9]. The most studied invasive species is *Aedes albopictus* (Skuse 1894), which is reported to possess greater heat and stress tolerance than other aedine species [10]. There are several examples of invasive mosquitoes moving or being transported between countries: In the 1990s and 2010s, the ranges of *Ae*. *japonicus* (Theobald 1901) and *Ae*. *koreicus* (Edwards 1917) expanded from East Asia to North America and Europe, respectively [11–21]. Additionally, in 2019, *Ae*. *flavopictus* (Skuse 1894) was detected for the first time in Europe [22]. Moreover, the spread of invasive mosquitos is not limited to species belonging to *Aedes*; *Anopheles* and *Culex* species have also been reported in locations outside their native ranges [23–25]. Many invasive mosquito species are known to have the ability to transmit infectious diseases, and there have been cases where endemic diseases have been transmitted between continents during the expansion of mosquito range [26, 27]. For example, *Anopheles arabiensis* (Patton 1905), the primary malaria vector in Africa, invaded Brazil from Senegal, resulting in a malaria pandemic that caused 16,000 deaths over a decade [28]. On the basis of previous studies, it can be inferred that if non-native mosquitoes are introduced, there is a potential for the concurrent emergence of mosquito-borne diseases.

In total, 59 species, 11 genera, and two subfamilies of mosquitoes have been recorded in the Republic of Korea (ROK) [29]. Among these, 14 species from four genera are considered potential vectors in the Korean Peninsula due to their reported ability to transmit diseases [30]. Except for endemic diseases (Japanese encephalitis and malaria), there have been no outbreaks of exogenous mosquito-borne diseases in the ROK to date.

The changing climate pattern in the Korean peninsula, which is causing a shift from temperate conditions to subtropical conditions, makes it possible for invasive species to become established in ROK, leading to the potential introduction of infectious diseases [31–34]. Jeju Island, located in the southernmost part of the ROK (central GPS coordinates: 33°23′N, 126°34′E), is expected to be the first region to report invasive mosquitoes. This island is the warmest region in the ROK, even in winter; furthermore, it is already classified as having a humid-subtropical climate [35]. Due to its distinct climate and geographic location, several nonnative insects have been reported recently on Jeju Island, including *Anoplophora horsfieldii* (Hope, 1843) (Coleoptera: Cerambycidae) in 2023 [36] and *Spodoptera frugiperda* (Smith, 1797) (Lepidoptera: Noctuidae) in 2019 [37]; These cases highlight the Jeju Island’s potential as a susceptible site for the invasion of non-native insect species.

In this study, we report the first record of *Ae*. (*Mucidus*) *laniger* (Wiedemann 1820) on Jeju Island, with taxonomic details and molecular evidence using phylogenetic method. Additionally, updates have been made on the mosquito species lists in Jeju Island and the taxonomic keys for genus *Aedes* in Korea. Furthermore, species distribution modeling analysis was conducted to explore the potential implications concerning this species and about other invasive mosquitoes on the Korean Peninsula.

## Methods

### Sample collection and identification

**Fig. 1:**
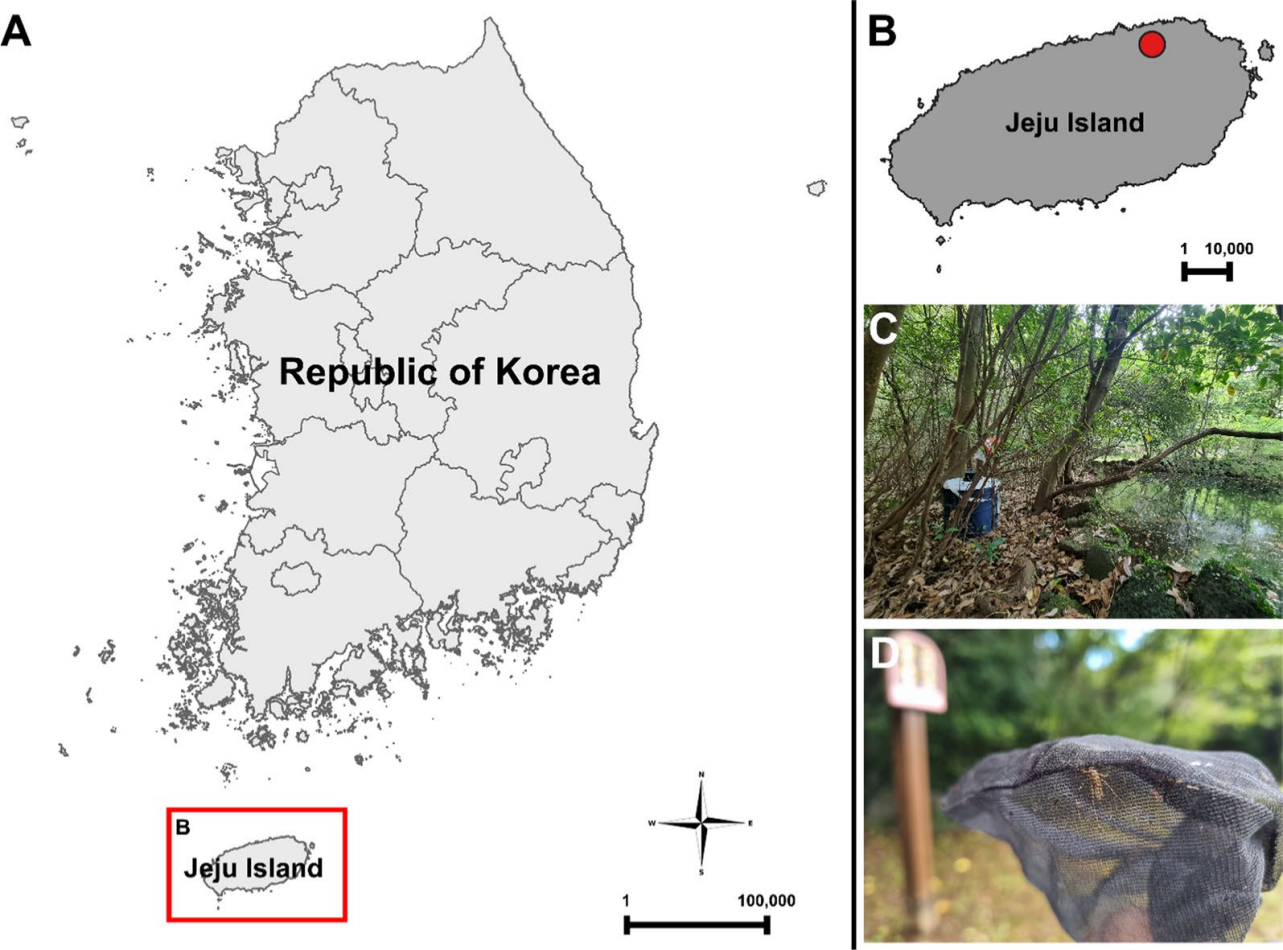
Collection site on Jeju Island. **A** Location of Jeju Island in the Republic of Korea, indicated by red box. **B** Location of the Dongbaek-dong wetland, indicated by red dot. **C** BG-sentinel trap used for collecting mosquitoes. **D** *Ae*. *laniger* specimen collected by using a BG-sentinel trap

Field surveys were carried out twice near Dongbaek-dong wetland, Jeju Island, in August 2023 (Fig. 1A–B). Adult specimens were collected using BG-sentinel^™^ (BGS) traps (Biogents, Regensburg, Germany) with BG–lure (lactic acid) and dry ice as attractants (Fig. 1C–D). The specimens were collected in highly humid and shaded forest near the wetland. There were many puddles around the wetland, and aquatic plants were abundant. Only two adult females were attracted by dry ice. The collected specimens were stored at −80 °C and subsequently identified using the taxonomic keys of Tanaka et al. [38] and Ree [39], which are for the mosquito species of East Asia (Korea and Japan). For the mosquitoes that could not be identified using these keys, the taxonomic keys of Southeast Asian mosquitoes were used [40–42]. Remaining specimen was used for molecular analysis.

### Taxonomy

The collected specimens were examined under a stereoscopic microscope (Leica M205 FCA, Germany). Photographs were taken with a Canon 90D camera with an MP-E 65 mm lens mounted on a Stackshot Macro Rail (Cognisys, Inc., USA). All the microphotographic layers were combined and retouched with Helicon Focus software v8.1.1 (HeliconSoft, Ltd., Ukraine); then, the merged image was edited using Photoshop v.2023 (Adobe). The morphological terms used in the descriptions follow those of Rattanarithikul et al. [40] and Harbach and Knight [43]. One female adult was pinned and deposited at Seoul National University in the Laboratory of Evolution and Phylogenomics (SNUE).

We follow Wilkerson et al. [44] for generic classification. *Aedes laniger* [45] belong to family Culicidae, subfamily Culicinae, genus *Aedes*, subgenus *Mucidus* [46].

#### Diagnosis

Female adult. See Tyson [41] and Mattingly [42] for morphological detailed characters of subgenus *Mucidus*. Characteristic scaling patterns: combinations of yellow, white, and brown, especially in mesonotum and legs, compared with other subgenera in *Aedes*. Maxillary palpus more than half length of proboscis. Scutum with highly modified. Wings with distinctive patterns; wing membrane pigmented in the region along the radiomedial cross–vein (rm), the base of vein R_4+5_, and the medio-cubital cross-vein (mcu).

#### Examined material

One female adult (SNUE), 19.VIII.2023, Jocheon-eup, Jeju–si, Jeju–do, Republic of Korea, 33°31′05.9′′N, 126°42′56.0′′E, BG-sentinel traps with dry ice, Woo Jun Bang; one female adult (SNUE), same data, but 30.VIII.2023, Woo Jun Bang and Heungmin Kim. In total, two female adults collected; one female pinned and another preserved in −80 ℃ freezer for further molecular studies.

### Molecular data acquisition

DNA was extracted from one grounded leg of the pinned specimen, using an OmniPrep™ for Tissue Kit (Cat. #786–395; G–Biosciences^®^, USA). A total of four partial gene regions—mitochondrial cytochrome c oxidase I (COI), internal transcribed spacer 2 (ITS2), large subunit ribosomal RNA (28S), and the nuclear protein-coding gene enolase—were used in the construction of a phylogenetic tree. Detailed information on the genes and primer sets used is provided in Table 1. All polymerase chain reaction (PCR) amplifications were conducted in a reaction mixture containing a total volume of 25 μL: 1 × PCR buffer, 0.4 μM each primer, 1.5 mM MgCl_2_, 0.2 mM each dNTP, 0.5 units of Taq DNA polymerase (R001AM; Takara Bio, Kusatsu, Shiga, Japan), and 1.0 ~ 2.0 ng of extracted DNA. The PCR procedure was as follows: 94 °C for 5 min for denaturation; 35 cycles of 94 °C for 30 s, 50–60 °C (50 °C for enolase and 28S, 52 °C for COI, and 60 °C for ITS2) for 30 s, and 72 °C for 30 s; with a final extension for 5 min at 72 °C. The products were visualized on 1.5% (wt/vol) agarose gels stained with Midori Green Advanced DNA Stain (Nippon Genetics Europe, Düren, Germany) and then sequenced in both directions by Bionics Corp. (Seoul, Republic of Korea). The sequencing data were assembled and trimmed using BioEdit software v7.2.6.1 [47] and were deposited in GenBank under the following accessions: *Ae*. *laniger*—PP097195 for COI, PP095639 for ITS2, and PP095638 for 28S; *Ae*. *hatorii*—PP095640 for 28S and PP215381 for enolase; *Ae*. *koreicus*—PP095641 for 28S and PP215377 for enolase; *Ae*. *albopictus*—PP215379 for enolase; *Ae*. *japonicus*—PP215378 for enolase; *Ae*. *nipponicus*—PP095642 for 28S; *Ae*. *togoi*—PP215380 for enolase. Additional sequences for the four gene regions of other species were downloaded from GenBank and used to construct the phylogenetic tree. Detailed information about the sequences used is provided in Additional file 1: Table S1.

**Table 1:** Four sets of primers used in this study

Marker	Primer name	Primer sequence (5ʹ to 3ʹ)	References
COI	LCO1490	GGTCAACAAATCATAAAGATATTGG	Folmer et al. [48]
	HCO2198	TAAACTTCAGGGTGACCAAAAAATCA	Folmer et al. [48]
ITS2	MS_ITS2_F	CTCGTGGATCGATGAAGACC	This study
	MS_ITS2_R	CTCGCAGCTACTCAGGGAAT	This study
28S	ms28S_F	CCGTGAGGGAAAGTTGAAAA	This study
	ms28S_R	TTTCCCCTGACTTCAACCTG	This study
Enolase	enoR2_F	AGRATYTGGTTGTACTTGGC	Soghigian et al. [49]
	enoF_R	ATGCAGGAGTTCATGATCCTG	Soghigian et al. [49]

### Molecular analysis

We performed a cross-check of the phylogenetic tree with the morphological identification results to assess the distinctiveness of the sample DNA from the subgenera recorded in the ROK, which include *Aedes*, *Aedimorphus*, *Bruceharrisonius*, *Collessius*, *Downsiomyia*, *Edwardsaedes*, *Hulecoeteomyia*, *Neomelaniconion*, *Ochlerotatus*, *Stegomyia*, and *Tanakaius*; except for *Hopkinsius*, no sequence data were registered in the National Center for Biotechnology Information (NCBI). We further sought to confirm the position of the sample within the subgenus *Mucidus*; our analyses were not intended to establish accurate and robust phylogenetic positions of all related subgenera of *Aedes*.

All the sequences were aligned using MAFFT v7.475 software [50] with the auto option, and subsequently manually trimmed in Aliview v. 1.26 [51]. The concatenated alignments were subsequently constructed with FASconCAT-G v1.02 software [52], and PartitionFinder 2 software [53] was used to determine the best partition scheme and substitution models according to codon position. We used IQ-tree v2.1.2 software to construct the maximum likelihood tree and applied the ultrafast option with 1000 replicates for bootstrapping [54, 55]. To provide additional branch support, the Shimodaira–Hasegawa-like approximate likelihood ratio test (SH-aLRT) was applied with 1000 replications to ensure that our data were not biased due to taxa with limited marker coverage [56]. We rooted the phylogeny along the branch leading to the genus *Psorophora*, which is known as the most ancestral clade of Aedini [57–60]. Finally, the constructed tree was visualized in iTol v5 [61] and enhanced for clarity using Photoshop v.2023 (Adobe).

### Species distribution modeling

Georeferenced *Ae*. *laniger* occurrence data were compiled from various resources, including previously published studies [41, 42, 45, 62–64], the Global Biodiversity Information Facility [65], which contains global occurrence records of thousands of species, and the iNaturalist database [66]. The keywords used to search the GBIF and iNaturalist databases were species “*Ae*. *laniger*” or “*Mucidus laniger*” and “*Ae*. *laniger*”, respectively.

Records within 20 km of each other were eliminated to reduce spatial autocorrelation, and duplicate records were removed, resulting in a total of 25 occurrence data points for further analysis. The longitudes and latitudes of the points were transformed to Universal Transverse Mercator (UTM) coordinates and projected to zone 52 N. In addition, 5000 pseudo-absence points were randomly generated around the distribution points to account for the sampling probability at each occurrence point.

Species distribution modeling was conducted using the R package BIOMOD2 to project the current and future distributions of *Ae. laniger* on the basis of occurrence data and pseudo-absence points [67, 68]. Climate data were acquired from WorldClim 2.1 using the R packages raster [69], rgeos [70], and rgdal [71]. Subsequently, ten algorithms were applied using the BIOMOD2 package: generalized linear model (GLM), generalized boosted model (GBM), classification tree analysis (CTA), artificial neural network (ANN), surface range envelope (SRE), flexible discriminant analysis (FDA), multiple adaptive regression splines (MARS), random forest (RF), extreme gradient boosting (XGBoost), and MaxEnt [72]. Each algorithm was executed five times, for a cumulative total of 150 runs to enhance statistical robustness. During these trials, 80% of the points were used for calibration, while the remaining 20% were reserved for evaluation. Model accuracy was evaluated by the true kill statistic (TSS), and scores that surpassed the threshold of 0.75 were considered indicative of satisfactory performance [73].

Finally, ensemble modeling was performed to optimize the prediction of *Ae*. *laniger* occurrence by combining several diverse models [74]. Furthermore, to predict the future distribution of *Ae. laniger*, three climate models included in the CMIP5 multimodel ensemble, considering two representative concentration pathways (RCP 2.6 and 8.5), were selected. RCP 2.6 anticipates a future characterized by ambitious climate policies, striving to constrain global warming to less than 2 °C. In contrast, RCP 8.5 portrays a worse scenario marked by high greenhouse gas emissions, lacking substantial climate mitigation measures. These climate models were subsequently applied to create projections of the distribution of *Ae. laniger* for 2050 and 2070. These projections were subsequently compared with the current distribution maps. The R code used in this study generally follows the methods proposed by Kim et al. [75] with minor modifications.

## Results

### Morphological identification

**Fig. 2:**
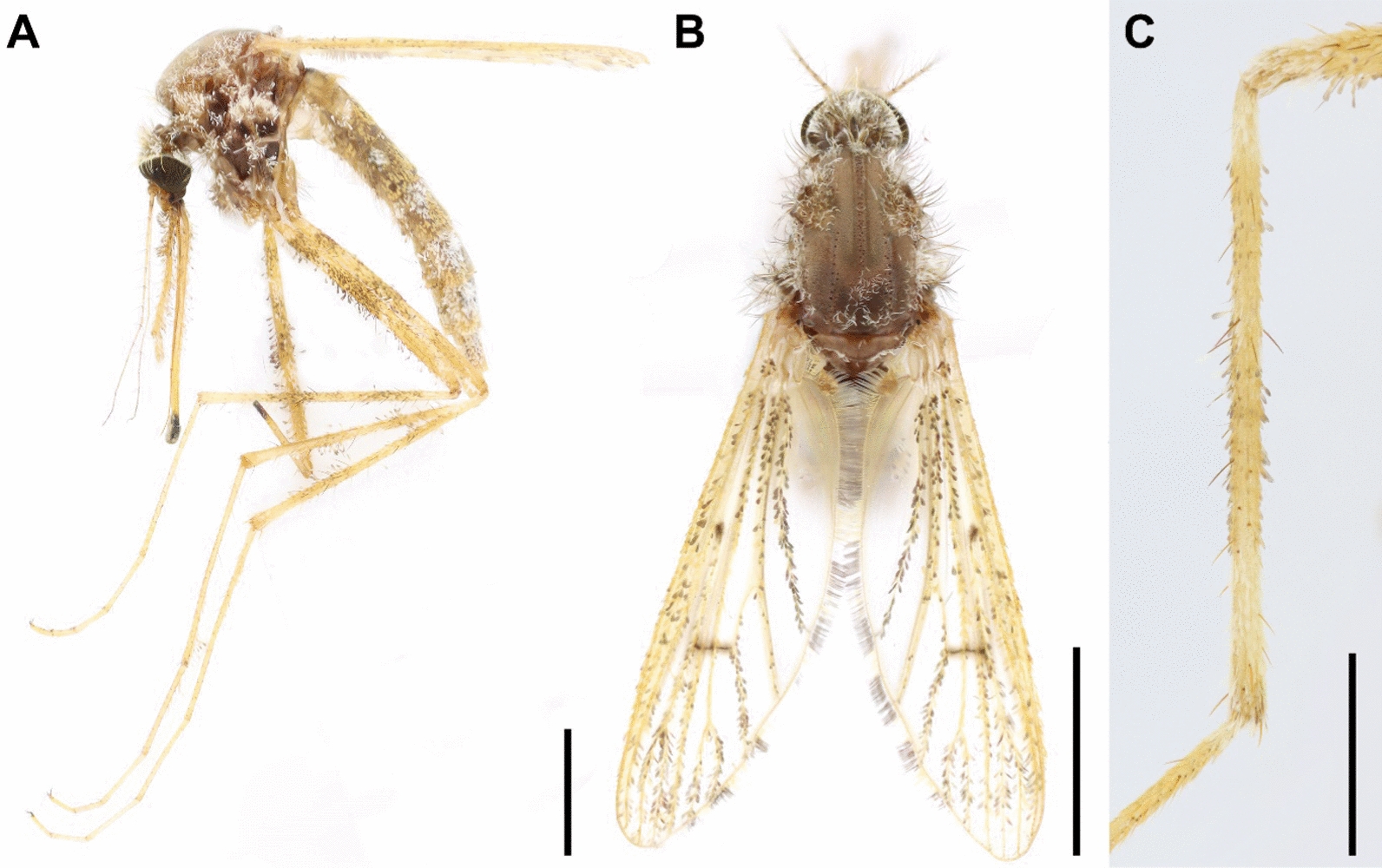
*Aedes laniger* adult female habitus in the **A** lateral view **B** and dorsal view and **C** fore tibia (scale bar 1.0 mm)

Female adult** (**Fig. 2A–C**)**. Habitus yellow-brownish (Fig. 2A). Scutum and scutellum with narrow white scales and setae mostly. Tergites mostly with yellowish scales at margin, and white scales mostly at median, but tergites VII–VIII with white scales extremely clothed **(**Fig. 2A–B**).** Wing veins mostly yellowish with pale, dark, and bicolored scales scattered accordingly; costa with numerous pale scales and patches; R_s_ with dark scales at base; from rm, the base of R_4+5_, to mcu with marked dark scales (Fig. 2B). Tarsi without bands, except on tarsomere I at base. Fore tibiae with narrowly white scales at base; largely white scales at apex about 0.3 the length of the tibiae **(**Fig. 2C**)**.

### Updated list of mosquito species on Jeju Island

A total of four species were captured using BG-sentinel traps at three trapping sites during field surveys in the Dongbaek-dong wetland, Jeju Island, in August 2023. The collected specimens belonged to two genera, *Armigeres* and *Aedes*, and were identified as *Armigeres subalbatus* (Coquillett, 1898), *Ae. albopictus*, *Ae. koreicus*, and *Ae. laniger*—a novel record for Jeju Island. Following the initial survey on Jeju Island conducted by Oh in 1957, this study provides an updated list, combining previous studies that includes 32 mosquito species belonging to 7 genera [76–85] (Table 2). We also updated the taxonomic keys for the genus *Aedes* in the ROK, including *Ae*. *laniger* (Additional file 2**)**.

**Table 2:** Updated checklist of mosquitoes found on Jeju Island, Republic of Korea: a total of 33 mosquito species belonging to 7 genera have been recorded in Jeju Island

Species	Oh [76]	Chun [77]	Lien [78]	5th PMU 1966–69	Tanaka et al. [38]	Lee [80]	Ko [81]	Kim [82]	Seo and Chung [83]	Chattejee [84]	Seo et al. [85]	This study	Remarks
*An*. *sinensis*	O	O	O	O		O	O	O	O	O	O*		
*An*. *lesteri*				O									
*An*. *lindesayi japonicus*			O		O	O	O						
*An*. *sineroides*		O		O	O	O	O		O	O			
*Ar*. *subalbatus*	O	O	O			O		O	O	O	O	O	
*Ae*. *albopictus*	O	O	O	O	O	O	O	O	O	O	O	O	
*Ae*. *flavopictus*					O	O							
*Ae*. *lineatopennis*				O									
*Ae*. *niponii*	O	O	O	O		O	O		O	O	O		
*Ae*. *hatorii*	O	O	O	O	O	O	O	O					
*Ae*. *japonicus*			O	O	O	O		O					
*Ae*. *koreicus*						O		O		O		O	
*Ae*. *nipponicus*								O					
*Ae*. *togoi*	O	O	O	O	O	O	O	O	O	O	O		
*Ae*. *dorsalis*										O			
*Ae*. *laniger*												O	**New record**
*Cx*. *bitaeniorhynchus*		O		O		O	O		O	O			
*Cx*. *hayashii*		O	O		O	O	O						
*Cx*. *infantulus*					O		O	O					
*Cx*. *inatomii*											O		
*Cx*. *kyotoensis*			O	O	O	O	O	O					
*Cx*. *sasai*								O					
*Cx*. *mimeticus*								O					
*Cx*. *orientalis*			O	O		O	O						
*Cx*. pipiens*	O	O	O	O	O	O	O	O	O	O	O		
*Cx*. *pseudovishnui*					O								
*Cx*. *tritaeniorhynchus*	O	O	O	O	O	O	O	O	O	O	O		
*Cx*. *vagans*		O		O		O		O	O	O			
*Lutzia fuscanus*			O			O	O	O					
*Lt*. *halifaxi*		O		O				O					
*Tripteroides bambusa*			O			O		O					
*Mansonia uniformis*									O	O			
Total number of species	8	13	15	16	14	19	15	18	11	13	8*	4	

Black asterisk indicates the species labeled as ‘sp.’ in the reference

### Molecular phylogeny

We employed a phylogenetic method to confirm a new record at the subgenus and species levels in the ROK. COI sequences, usually used for DNA barcoding, are not available from the NCBI for *Ae*. *laniger*. For this reason, multigene molecular phylogenetic analysis was used to assess the phylogenetic position of *Ae. laniger* within the subgenus *Mucidus* with existing data for *Ae*. *alternans* (Westwood 1836) and *Ae*. *sudanensis* (Theobald, 1908). In addition, we aimed to investigate whether the collected specimen belong to 11 subgenera recorded in the ROK.

On the basis of the four molecular markers, the concatenated matrix consisted of 2741 nucleotide sequences from 42 species of *Aedes*, and the five best partitioning schemes (GTR + G, GTR + I + G, TVM + I + G, TIM + I + G, and TVM + I) were assigned to eight subsets.

**Fig. 3:**
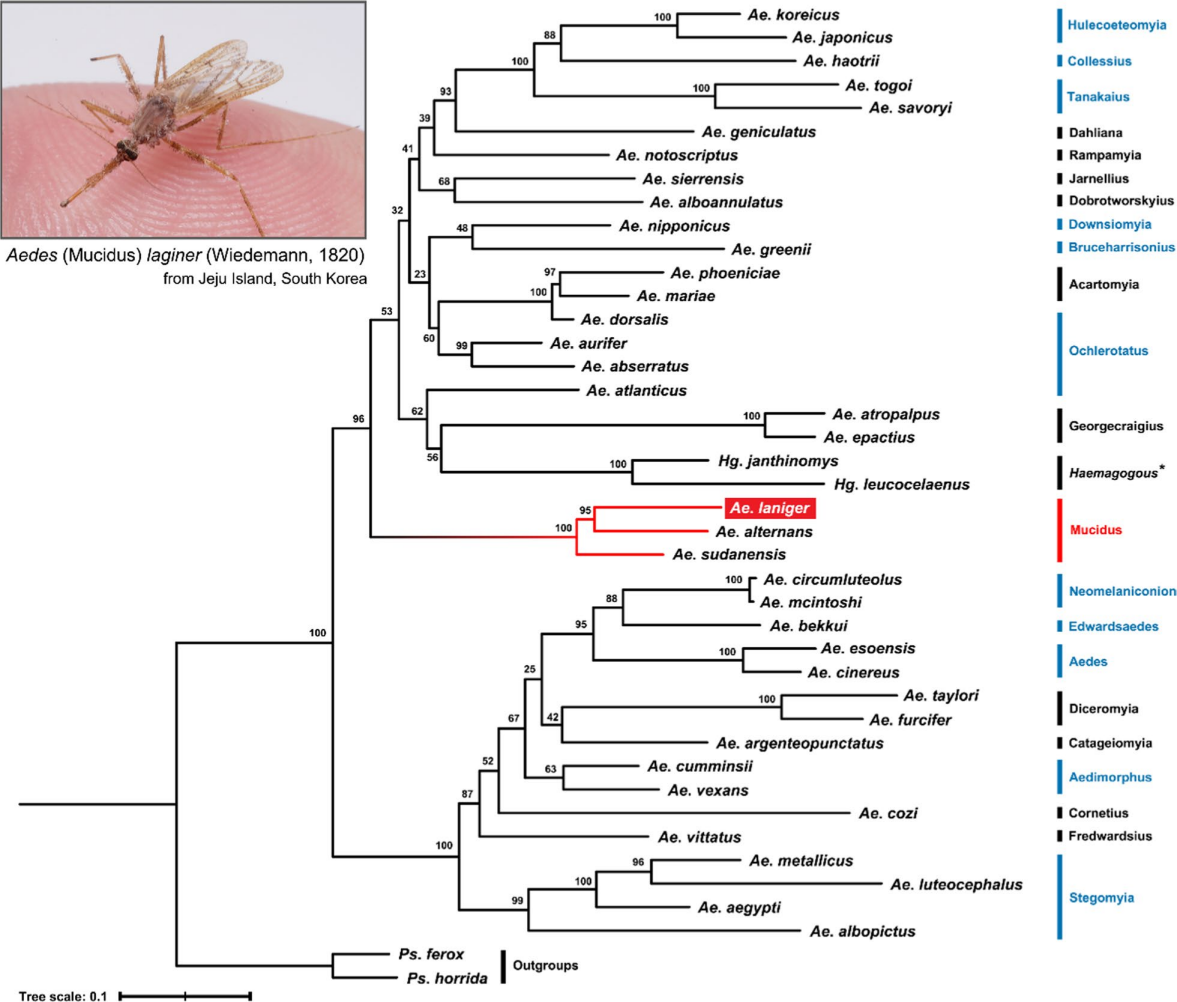
Maximum likelihood phylogeny of the Aedini species, comprising 3 genera, 22 subgenera, and 40 species (including 11 belonging to subgenera present in Korea). The tree is based on four marker genes (COI, ITS2, 28S, and enolase) and includes 11 newly sequenced data, with two species of *Psorophora* for outgroups. The substitution models were selected using PartitionFinder2, and 2000 bootstrap replicates were performed with 1000 SH-aLRT tests. The black asterisk indicates a genus, not a subgenus. The blue texts and lines indicate the recorded subgenera in the ROK. The red texts and lines indicate the subgenus *Mucidus*, which showed the key points of this study

The phylogenetic tree showed that the collected specimen was not assigned to the clades of subgenera recorded in the ROK, but rather was grouped with *Ae*. *alternans* and *Ae*. *sudanensis*, which are species of subgenus *Mucidus* with high support values (SH-aLRT > 85, Ultra Fast Bootstrap > 95) (Fig. 3). Furthermore, the subgenera recorded in the ROK each formed a distinct clade, and two paraphyletic clades were found in the genus *Aedes*, consistent with numerous studies of mosquito phylogenetics based on morphological traits or molecular phylogeny [49, 57, 58].

We initially identified the collected specimen as *Ae*. *laniger* through the morphological keys at the species level, and molecular analysis also showed that the specimen forms a distinct clade with the subgenus *Mucidus*, which means a new record within the subgenus *Mucidus* and the species in the ROK. Finally, the outcomes of this study confirmed the concordance between morphological and molecular species identification results.

### Species distribution modelling

**Fig. 4:**
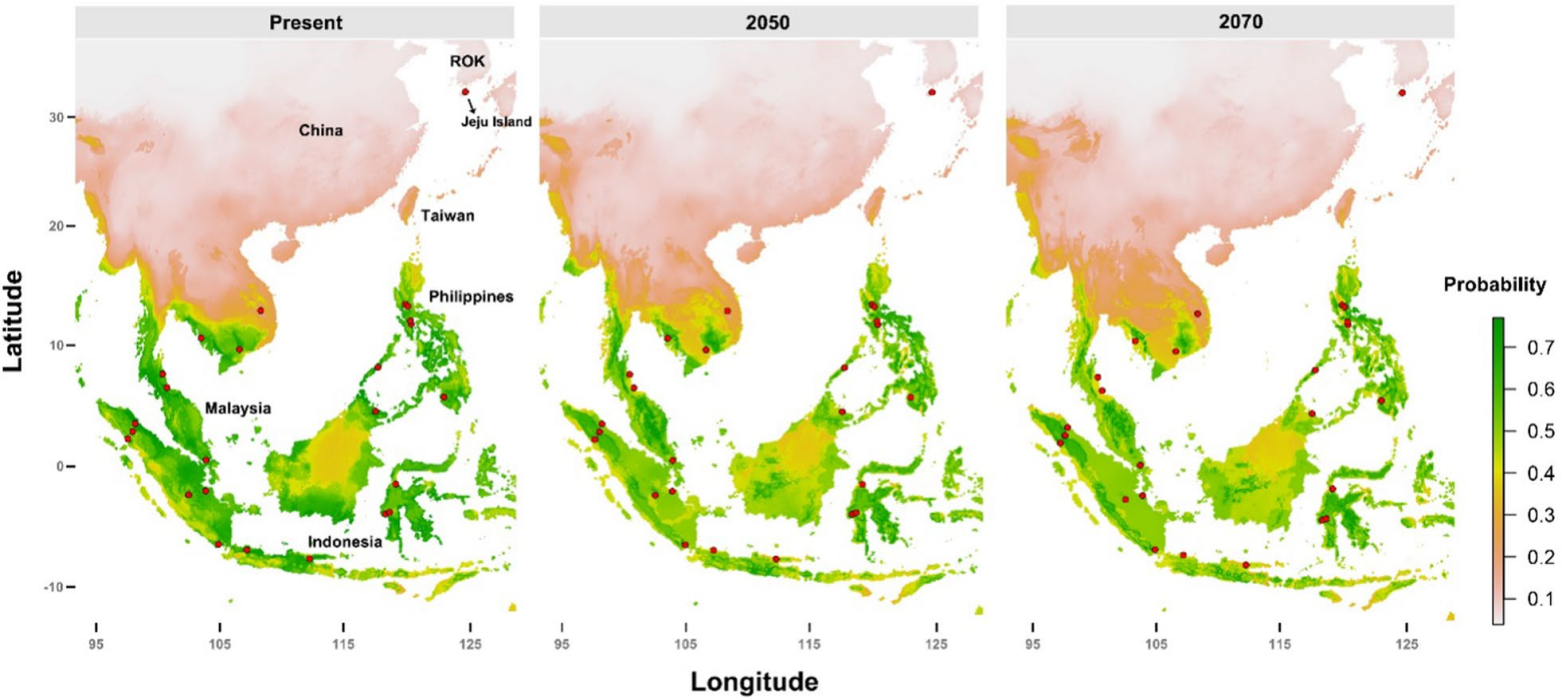
Projected distributions of *Ae*. *laniger* under the current climate conditions and in 2050 and 2070 based on ensemble species distribution modeling. The closer the color is to green, the higher the probability for species to inhabit the corresponding region. The red circles indicate occurrence records, and the plots are presented as “weighted mean results” generated using the R package BIOMOD2

The results of the species distribution models suggest that within Southeast Asia, *Ae*. *laniger* is most likely to be found in Indonesia, Malaysia, and the Philippines, all of which were identified as areas with highly suitable climate spaces for this species (Fig. 4). In contrast, the modeling results indicate that among East Asian countries, including South Korea and China, Jeju Island has suboptimal conditions for this species.

Models were created on the basis of climate modeling data for 2050 and 2070 considering RCP2.6 and RCP8.5 to assess the potential for *Ae*. *laniger* to spread beyond its current distribution. The results of these models revealed that the suitable climate space of *Ae. laniger* could increase, extending from Taiwan to southern Myanmar and the northern regions of Thailand. These results suggest the potential for the expansion of *Ae*. *laniger* habitat by 2050 and 2070 in response to climate change.

In summary, we identified *Ae*. *laniger* on Jeju Island through morphological and molecular analysis. Subsequently, species distribution modeling was performed under both current and future climate scenarios. The results indicate a subtle expansion of *Ae*. *laniger* from Taiwan to the Indomalayan regions, attributed to ongoing climate changes.

## Discussion

On the basis of morphological identification and molecular analysis, the collected specimen was identified as *Ae. laniger*, a Southeast Asian mosquito species, representing a new record in the ROK. We readily identified the collected specimen as belonging to the subgenus *Mucidus* due to their distinctive wing pigmentations and the scaling patterns observed across their bodies. Furthermore, molecular phylogeny also supported the position of the specimens within the *Mucidus* clade, distinct from previously recorded subgenera in Korea. These results confirmed the presence of a new nonnative subgenus and species in the ROK. Therefore, we report a total of 33 species, 7 genera, and 2 subfamilies recorded in Jeju Island, and 60 species, 11 genera, and two subfamilies of mosquitoes recorded in the ROK. It has been confirmed that the species is distributed throughout Cambodia, India, Indonesia, Malaysia, the Philippines, Singapore, Thailand, Vietnam, and Jeju Island in Korea.

This study also suggests that even in the absence of COI sequence, commonly used as a DNA barcode, the application of multigene phylogeny could facilitate the detection of non-native species at the genus or subgenus levels for pest control purposes. Specifically, for poorly studied species with limited morphological and molecular data, the workflow used in this study enables the identification of non-native species without relying on COI sequences.

*Ae. laniger* occurs in subtropical or tropical regions of Southeast Asia, Australia, and Africa; it is not native to East Asia [38, 40–42]. For this perspective, the occurrence of *Ae. laniger* suggests that subtropical or tropical mosquitoes have the potential to expand their habitats to the ROK. Lee et al. [86] also reported that the strain of *Ae. albopictus* on Jeju Island is genetically closely related to specimens from Southeast Asia, which have a high transmission capability for dengue fever, thereby raising concerns regarding its potential risks.

The confirmation of Southeast Asian mosquito species on Jeju Island aligns with predictions reported in prior studies, as well as with reports of other non-native species. However, the results of the species distribution modeling do not indicate a strong propensity for *Ae*. *laniger* to inhabit the Korean Peninsula; this discrepancy may be due to the lack of species occurrence data. Nevertheless, on the basis of the available data, a discernible pattern emerged, indicating that *Ae. laniger* is likely to be predominantly found in coastal areas or on islands characterized by high humidity and temperature. Given these characteristics and the recognition that Jeju Island has coastal areas with a subtropical climate, it is plausible that *Ae. laniger* could establish breeding populations on this environment.

Furthermore, there was a recent outbreak of love bugs, *Plecia longiforceps*, in the ROK [87], and the nonnative species *A*. *horsfieldii* was identified on Jeju Island in 2019 [36]; these species are distributed mainly in southern China, Taiwan, and the Okinawa archipelago. These cases suggest the potential for provisional or temporary spread of nonnative species to the ROK. Jung et al. [88] also suggested the potential emergence of *Ae*. *aegypti* in the coastal areas of Jeju Island starting in 2040 due to climate change, using species distribution modeling.

In addition to climate factors, invasion can be influenced by wind. In the ROK, *Nilaparvata lugens* and *Spodoptera frugiperda* are known to spread to the island by riding westerlies in the spring or tropical cyclones in the summer [37, 89, 90]. Typhoons typically affect the Korean Peninsula to the greatest extent in July and August [91]. The two specimens captured in this study were collected in August, suggesting that their presence on the island may have been influenced by wind patterns in that period. Studies have shown that mosquitoes can spread through various means, including winds, airplanes, or watercraft, indicating the potential for provisional spread of the species [3, 6].

Notably, Jeju Island has also been identified as a bridgehead for the introduction of tropical or subtropical insects into the Korean Peninsula [37, 92, 93]. The results of this study and those of other investigations suggest that there is potential for the continued presence of Southeast Asian mosquito species on Jeju Island in the future.

The species distribution models applied in this study did not yield strong predictions for *Ae*. *laniger* to East Asia. The constraints imposed by the limited availability of occurrence data, along with the concentration of coordinates in Southeast Asia, probably led to nonsignificant outcomes [94, 95]. Given the tropical and subtropical climates in Taiwan and the Okinawa archipelago, further investigations to confirm the habitat of *Ae. laniger* in these regions would enhance the resolution of our analysis.

This study was confined to a limited area of Jeju Island, and further investigations across various regions are needed to expand our understanding of the broader ecological implications and potential range of *Ae*. *laniger*. The Dongbaek-dong wetland is characterized by a distinctive hot and humid environment, with numerous swampy areas, flood pools, and temporary pools, consistent with the habitat characteristics of *Ae. laniger* [41, 42]. The presence of this species in the Dongbaek-dong wetland suggests its potential to inhabit similar habitats on Jeju Island.

Additionally, it remains unclear whether the presence of *Ae. laniger* on Jeju Island is temporary or if it has established a breeding population on the island. To determine whether permanent populations of *Ae*. *laniger* have been established, multiyear monitoring surveys seem to be needed. Most subtropical or tropical mosquitoes face challenges in surviving cold winters; therefore, it is recommended that both adult and larval collections be conducted in subsequent monitoring surveys.

The key outcome of this study is the initial identification of mosquitoes adapted to the Southeast Asian climate in the ROK. Despite the uncertainty of the vector competence of *Ae. laniger* or other species of subgenus *Mucidus* [41, 96], this finding is significant, as it indicates the potential for future occurrences of vector species that are native to Southeast Asia, capable of transmitting the viruses responsible for dengue fever and Zika virus on the Korean Peninsula.

## Conclusions

Invasive species from tropical or subtropical regions are becoming common on Jeju Island, and the results of this study were consistent with that pattern. This study represents the initial confirmation of a Southeast Asian mosquito species in Korea and indicates the potential for the spread of other vector mosquitoes from subtropical areas in the future. The proposed workflow in this study also showed that, even without the COI sequence, detection of non-native or pest species at the genus or subgenus is possible through the application of a multigene phylogeny approach with a limited number of markers.

## Supplementary Information

Additional file 1. Table S1. The gene dataset and sequences used for the phylogenetic analysis.

Additional file 2. Taxonomic key to the species of the genus Aedes in the Republic of Korea.

## Data Availability

The datasets produced in this study are incorporated in the article (Additional file 1), and the sequences (PP095638, PP095639, PP095640, PP095641, PP095642, PP097195, PP215377, PP215378, PP215379, PP215380, PP215381) utilized have been deposited in the NCBI database.

## Abbreviations

ROK: Republic of Korea
SNUE: Seoul National University in the Laboratory of Evolution and Phylogenomics
COI: Cytochrome c oxidase subunit I
ITS2: Internal transcribed spacer 2
NCBI: National Center for Biotechnology Information
UTM: Universal Transverse Mercator
SH-aLRT: Shimodaira–Hasegawa approximate likelihood ratio test
UFB: ultrafast bootstrap
