# Supplementary material for: Insights from multigene analysis: first report of a Southeast Asian Mosquito, Aedes (Mucidus) laniger (Diptera: Culicidae) on Jeju Island from Korea
Source: Parasit Vectors. 2024 Sep 12;17:386. doi: 10.1186/s13071-024-06373-8 (PMC11395179; doi:10.1186/s13071-024-06373-8)
Supplement: Supplementary file 2 — Additional file 2. Taxonomic key to the species of the genus Aedes in the Republic of Korea. [file 13071_2024_6373_MOESM2_ESM.pdf]

Key to the species of genus *Aedes* in Republic of Korea (based on Ree 2003)

Adult female

1. Maxillary palpi longer than half length of proboscis, wings with bicolorous fringes and distinctive spots along the veins … ***Aedes laniger***

- Maxillary palpi much shorter than proboscis; wings mostly with unicolorous fringes and normal scaling patterns … 2

2. Tarsi without bands … 3

- Tarsi with bands … 7

3. Scutellum with broad appressed scales … 4

- Scutellum with narrow curved scales … 5

4. Scutellum with broad dark scales; scutum with a pair of large anterolateral white patches … ***Aedes nipponicus***

- Scutellum with broad white scales; scutum dark without patches … ***Aedes alboscutellatus***

5. Scutum yellowish brown without stripes … ***Aedes esoensis***

- Scutum dark with stripes or bands … 6

6. Scutum with a pair of sublateral golden yellow patches forming line … ***Aedes lineatopennis***

- Scutum with 5 stripes of golden white scales; a median stripe bifurcating posteriorly and a pair of dorsocentral stripes almost connected posterior margin, and connected a pair of sutural stripes … ***Aedes oreophilus***

7. Tarsomere V of hind legs entirely white … 8

- Tarsomere V of hind legs black with/without basal bands … 12

8. Scutum with brown narrow median stripe and two pale broad submedian stripes forking posteriorly … ***Aedes dorsalis***

- Scutum mostly covered with black scales or patches (stripes) … 9

9. Scutum with two large triangular patches on fossa, a small anteromedian spot, a small

prescutellar spot, and two short posterior dorsocentral stripes, all with white scales … ***Aedes chemulpoensis***

- Scutum with stripes … 10

10. Scutum with a median stripe and bifurcated posteriorly, a pair of long arched lateral stripes … ***Aedes galloisi***

- Scutum with anterior median stripe and short 4 posterior submedian stripes with white scales … 11

11. Yellowish narrow curved sparsely scales at lateral margin of scutum just above the wing root … ***Aedes flavopictus***

- Silvery white broad compressed scales at lateral margin of scutum just above the wing root … ***Aedes albopictus***

12. Scutum with yellowish brown scales without stripes or patches; eight abdominal segment narrow and completely retractile … 13

- Scutum with patches or stripes; eight abdominal segment not completely retractile … 14

13. Abdominal tergite I with dark scales in middle; tergites II-VII with large laterobasal pale patches only; wings not speckled … ***Aedes bekkui***

- Abdominal tergite I with pale scales in middle; tergites II-VII with basal pale bands, laterobasal patches and apical median patches usually on tergites II, VI and VII; wings speckled on subcosta and vein 1 or on costa basally… ***Aedes nipponii***

14. Hind tarsomeres I-III with pale bands only at base … 15

- Hind tarsomeres I-III with pale bands along the joints … 16

15. Hinad tarsomere IV with a white band at base; subspiracular area with broad white scales … ***Aedes koreicus***

- Hind tarsomere IV without band; subspiracular area without scales … ***Aedes japonicus***

16. Scutum mostly covered with white or whitish scales anteriorly … ***Aedes seoulensis***

- Scutum mostly dark with several pale scales arranged in more or less definite lines… 17

17. Maxillary palpi entirely dark; scutum with golden yellowish patches connected by short stripes; vertex with pale yellow erect forked scales mixed with others … ***Aedes alektorovi***

- Maxillary palpi pale at apex; scutum dark or dark brown with yellowish brown scales forming pairs of median, dorsocentral and lateral stripes … 18

18. Hind tarsomere V entirely white; fore tarsomere IV-V entirely dark … ***Aedes hatorii***

- Hind tarsomere V dark at apex; fore tarsomere IV-V with pale bands along the joints … ***Aedes togoi***
